# Case report: Multidisciplinary collaboration in diagnosis and treatment of child gaucher disease

**DOI:** 10.3389/fped.2023.1057574

**Published:** 2023-03-30

**Authors:** Jianfang Zhu, Yuxiao Sun, Weiyan Zheng, Chunlin Wang

**Affiliations:** ^1^ Department of Pediatrics, The First Affiliated Hospital, Zhejiang University School of Medicine, Hangzhou, China; ^2^ Department of Hematology, The First Affiliated Hospital, Zhejiang University School of Medicine, Hangzhou, China

**Keywords:** gaucher disease, glucocerebrosidase, multidisciplinary collaboration, splenomegaly, case report

## Abstract

Gaucher disease (GD) is an inherited lysosomal storage disease caused by mutations in the glucocerebrosidase gene. The decrease of glucocerebrosidase activity in lysosomes results in the accumulation of its substrate glucocerebroside in the lysosomes of macrophages in organs such as the liver, spleen, bones, lungs, brain and eyes, and the formation of typical storage cells, namely “Gaucher cells”, leading to lesions in the affected tissues and organs. Hepatosplenomegaly, bone pain, cytopenia, neurological symptoms, and other systemic manifestations are common in clinical practice. Most pediatric patients have severe symptoms. Early diagnosis and treatment are crucial to improve the curative effect and prognosis. However, due to the low incidence of this disease, multi-system involvement in patients, and diverse clinical manifestations, multidisciplinary teamwork is needed for comprehensive evaluation, diagnosis and treatment. In this study, we reported 2 cases of different types of GD who were diagnosed, treated and followed up by multidisciplinary collaboration in infancy.

## 1. Introduction

GD is a clinically rare single-gene autosomal recessive metabolic disorder. Due to the mutation of the glucocerebrosidase (GBA) gene, the activity of GBA in the lysosomes is reduced, which causes its substrate glucocerebroside to be stored in the lysosomes of macrophages in organs such as the liver, spleen, bones, lungs, brain and eyes to form “Gaucher cells”. The clinical manifestations are usually hepatosplenomegaly, bone pain, anemia, thrombocytopenia, neurological symptoms, and other systemic manifestations, which may progressively worsen during the course of the disease. There are three types of GD according to whether the nervous system is involved and the rate of disease progression (1–4). (1) Type I has historically been characterized by the absence of neurological involvement. Nevertheles, the prevalence of peripheral neuropathy in adults with type I has been reported to be higher than that in the general population (5). Evidence suggests that central nervous system (CNS) involvement may also occur in patients with type I (6, 7). Two third of type I develop the disease in childhood, and the severity of symptoms varies greatly. (2) Type II is an acute neuropathic type with extensive and severe visceral involvement, which usually develops within the first year after birth, and most patients die at the age of 2 to 4 years. (3) Type III is a subacute or chronic neuropathic type, which often develops in childhood. The early manifestations are similar to those of type I. The neurological symptoms of varying degrees of severity gradually appear with slow disease progression. In addition, there are rare subtypes such as perinatal lethal type and cardiovascular type (8).

The clinical symptoms and signs of GD are not characteristic, which can be insidiously in children or adults at different ages. Many medical diseases, such as leukemia, lymphoma, Niemanpick disease, and Langerhans cell histiocytosis, have similar manifestations to GD, which can easily lead to delayed diagnosis. A survey data from multiple countries (9) showed that many patients with GD experienced delays in diagnosis and misdiagnosis, and nearly 1/6 of the patients took more than 7 years to be diagnosed. Nevertheless, the two cases in this study were both diagnosed within six months of the onset of clinical symptoms. It is believed that with the continuous improvement of the understanding of rare diseases and the continuous development of diagnostic techniques, rare diseases including GD will be diagnosed and treated timely and accurately (10). In this study, we reported 2 cases of different types of GD who were diagnosed, evaluated, treated and followed up by multidisciplinary collaboration in infancy.

## 2. Patient information

### 2.1. Diagnostic assessment

#### 2.1.1. Involvement of the digestive system (gastroenterology)

Hepatosplenomegaly, especially splenomegaly, is the main manifestation of digestive system involvement in GD, which is often accompanied by hypersplenism, sometimes giant spleen, splenic infarction, and splenic rupture (11). Hepatosplenomegaly may be asymptomatic or manifest as anorexia, abdominal distension, abdominal discomfort, or dull epigastric pain, and rarely, acute abdominal pain due to splenic infarction. In this study, both children firstly visited the department of gastroenterology because of abdominal distension. Case 1 was a female child, aged 1 year and 7 months (date of birth 2018.9, G1P1, full-term natural delivery, birth weight 3.2 kg, no history of asphyxia rescue). She went to the doctor because of “abdominal distension and appetite reduction for 3 months”. Physical examination findings were as follows: abdominal distention, prominent on the left side; tough and sharp edged liver 2 cm below the ribs; splenomegaly, 3 cm below the umbilicus, with the lower margin close to the groin, hard in texture, no obvious swelling on the surface; normal liver function and coagulation function, negative for hepatotropic virus, normal serum copper, ceruloplasmin, ferritin, transferrin saturation and total iron binding capacity. Abdominal ultrasound showed that the right hepatic lobe was 2.2 cm below the ribs with the oblique diameter of 8.3 cm, and there were a smooth and complete capsule, uniform intrahepatic echoes, no obvious abnormal echoes, no obvious dilation of the intrahepatic bile duct, and clear intrahepatic vascular orientation. The spleen was half-moon-shaped and evenly hypoechoic, with a smooth and complete capsule, intercostal thickness of 4.6 cm, the lower margin 3.5 cm below the umbilicus, and 3.7 cm across the midline at the right margin. The gallbladder and pancreas were normal. Enhanced CT of the whole abdomen also indicated hepatosplenomegaly. Case 2 was a male child, aged 1 year (date of birth 2019.4, G1P1, full-term cesarean section, birth weight 3.6 kg, no history of asphyxia rescue). He went to the doctor because of “abdominal distension and appetite reduction for 1 month”, with abdominal distention and obvious enlargement of liver and spleen. Auxiliary examination revealed normal liver function and coagulation function, negative for hepatotropic virus, and normal serum copper, ceruloplasmin, ferritin, transferrin saturation and total iron binding capacity. Abdominal ultrasound showed that the right hepatic lobe was 2.8 cm below the ribs with the oblique diameter of 7.8 cm, and the size of the left liver was about 5.0*6.6 cm. The spleen was large in shape with thickness of 3.2 cm, 5.4 cm below the ribs, with a smooth and complete outline, and the even and delicate echoes of spleen parenchyma. The gallbladder and pancreas were normal. Abdominal CT revealed hepatomegaly, splenomegaly and enlarged hilar lymph nodes. After treatment with enzyme replacement therapy (ERT), the hepatosplenomegaly of both children was significantly improved, and the liver function was within the normal range.

#### 2.1.2. Involvement of the hematologic system (hematology)

**Figure 1:**
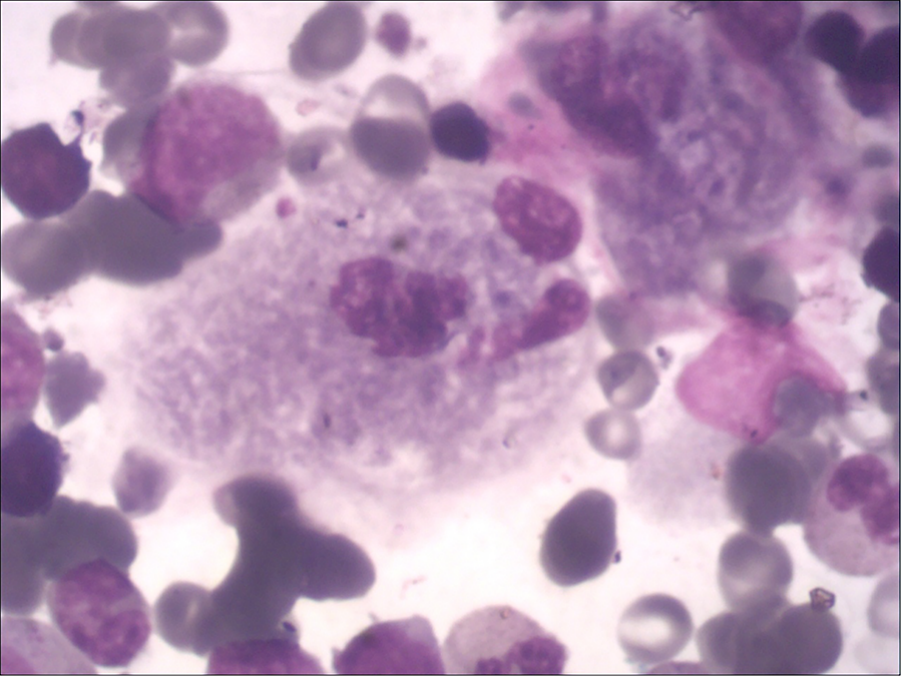
Gaucher cells in the bone marrow, with large or giant cell bodies, small nuclei, nuclear deformities, multinucleation, and dense chromatin. The cytoplasm is abundant and arranged in a gray-blue “onion skin” pattern.

The main manifestation are cytopenia (anemia, thrombocytopenia, leukopenia, and agranulocytosis may occur alone or simultaneously) and bleeding tendency (increased bleeding tendency in GD patients is associated with thrombocytopenia, abnormal coagulation and platelet function defects). Two children in this study had mild anemia and thrombocytopenia (case 1: 96 g/L, 71 × 10E9/l; case 2: 101 g/L, 98 × 10E9/L), and the leukocytes and coagulation function were within the normal range. Anemia and thrombocytopenia gradually recovered within 6 months of ERT in both two patients. Bone marrow biopsy was performed in both children by the department of hematology, and Gaucher cells were found in both cases (Figure 1). The enzyme activity test showed that *β*-GBA in case 1 was 3.1 nmol/mg·h, and *β*-GBA in case 2 was 1.2 nmol/mg·h, both significantly lower than the normal reference range (6.56∼55.1 nmol/mg·h). GD genetic testing reported that case 1 was a homozygous mutation of GBA gene c.1448 T > C (p.L483P), and case 2 was a compound heterozygous mutation [c.475C > T (p.R159W) heterozygous mutation (from mother); c.1448 T > C (L483P) heterozygous mutation (from father)]. The above mutation sites are all known pathogenic mutations (12).

#### 2.1.3. Involvement of the skeletal system (orthopedics)

**Figure 2:**
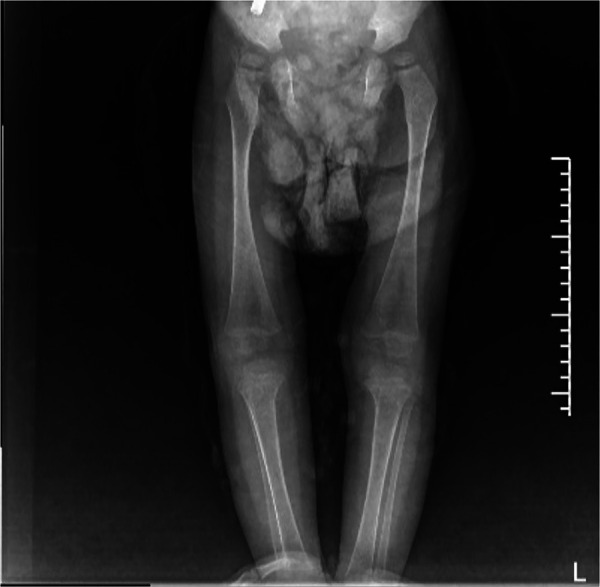
Signs of GD on both sides of the femur and tibia on October 21, 2021: enlarged and flask-shaped distal femur and proximal tibia on both sides, widened bone marrow cavity, and reduced bone density. No abnormal signs were observed in soft tissue.

GD can involve the whole-body bone with varying degrees of severity. The pathological change is the deposition of Gaucher cells in the bone, destroying and replacing the normal bone tissue. The affected parts mainly include the early lumbar spine, the metaphysis of long bones, the diaphysis, and the epiphysis in the middle and late stages. Patients with osteonecrosis often have acute/chronic bone pain, and may develop sudden local pain, swelling, or fever, and even aseptic osteomyelitis (13). In this study, both children had bone destruction. In case 1, bone imaging revealed low density lesions in the medial cortex of the right humerus and uneven bone density in both iliac bones. The remaining long bones of the limbs and spine were in normal shape, with continuous bone cortex and no abnormal changes in bone density. In case 2, the bone imaging examination showed that the left radius was unnatural, with local thickening of the cortical bone, and the x-rays of the remaining long bones of the limbs showed no obvious abnormality. The long bone destruction of the limbs occurred during the treatment and follow-up (Figure 2).

#### 2.1.4. Involvement of the endocrine system (endocrinology)

Endocrine involvement in GD mainly involves abnormal growth and development, bone metabolism, nutritional status and glucose metabolism. GD patients with childhood/adolescent onset are prone to growth retardation and a higher risk of malnutrition (14). In this study, 2 children were evaluated by the department of endocrinology at the beginning of the disease. Case 1 was severely growth retardation and development and malnutrition [height 71 cm (-3.21SDS), weight 7.5 kg (-2.8SDS); patent anterior fontanelle, 0.5*0.5 CM, head circumference 44 cm, chest circumference 46 cm, subcutaneous fat 0.3 CM] with normal bone metabolism and glucose metabolism indexes. Case 2 has moderate growth retardation and malnutrition [height 70 cm (-2.41SDS), weight 8 kg (-1.95SDS); patent bregma, 1.0*1.0 CM, head circumference 43 cm, bust circumference 45 cm, subcutaneous fat 0.4 CM] with normal bone metabolism and glucose metabolism indexes. After the primary disease treatment, nutritional guidance was given on carbohydrates (carbohydrates with low glycemic index are preferred, sugary foods are avoided, and foods rich in dietary fiber are adequately consumed), protein (referring to the recommended intake of protein for the same age, based on high-quality proteins such as animal and soy proteins), fat (appropriate intake of fat, limited intake of saturated fatty acids, and increasing the intake ratio of monounsaturated fatty acids and polyunsaturated fatty acids), minerals and vitamins (supplementation of vitamin D and calcium according to monitoring indicators). At 2.3 years of follow-up, case 1 (3 years and 11 months) was evaluated as mild growth retardation [height 96 cm (−1.67SDS), weight 13 kg (−1.73SDS); head circumference 48 cm, chest circumference 52 cm, subcutaneous fat 0.5 cm] with normal bone metabolism and glucose metabolism indexes. Case 2 (3 years and 3 months) was evaluated as severely retarded in growth and development [height 82 cm (−4.33SDS), weight 9 kg (−3.91SDS); head circumference 45 cm, chest circumference 48 cm, subcutaneous fat 0.3CM].

#### 2.1.5. Involvement of the nervous system (neurology)

**Figure 3:**
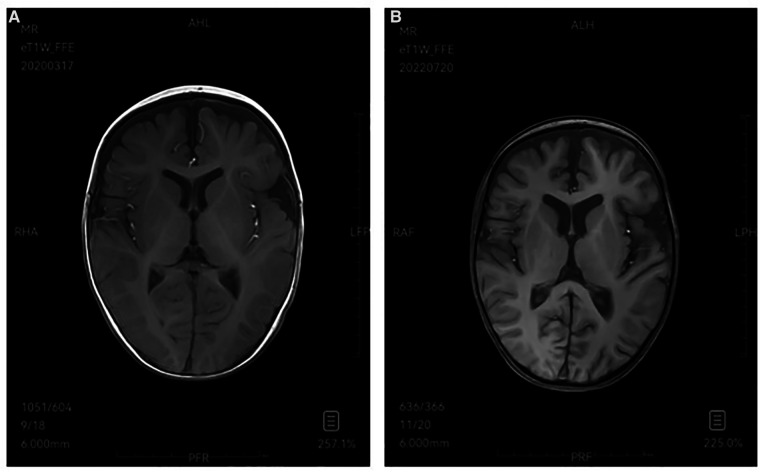
(**A**) 2020-3 MRI-T1W image demonstrates bilateral frontotemporal extracerebral space widening. (**B**) 2022-7 MRI-T1W image shows brain atrophy.

Type I GD has no significant manifestation of primary central nervous system involvement in childhood, while type II and type III GD are neuropathic type associated with nervous system involvement. Type II is an acute neuropathic type, with onset from the neonatal period to infancy, mainly manifesting as early-onset and rapidly progressive neurological involvement, such as bilateral fixed strabismus, oculomotor nerve palsy, sucking and swallowing difficulties, as well as epilepsy, opisthotonos, and cognitive disorders. Patients with type II GD usually die at the age of 2 to 4 years (15). Type III is chronic or subacute neuropathy type, and the early manifestation is similar to type I. The neurological symptoms of varying degrees of severity gradually appear with slow disease progression, even remain mild throughout their lives. Patients may have oculomotor involvement, horizontal eye movement disorders and ataxia, epilepsy, myoclonic seizures, developmental delay, intellectual disability (15, 16). There are usually electroencephalogram abnormalities, most of which are characterized by slow wave background and interictal epileptiform discharges (sharp wave, spike wave, multiple spike wave, spike-slow complex wave, etc.) (17). In this study, both children were evaluated by the department of neurology. Case 1 had no positive signs in nervous system examination, no abnormality in head MRI and EEG, and no nervous system involvement, and was diagnosed as type I GD. Case 2 had onset in infancy with delayed language and motor development, and early neurological symptoms (initially shaking limbs during sleep, then rapidly progressing to tonic convulsions and frequent seizures). Initial evaluation of head MRI showed bilateral frontotemporal extracerebral space widening (Figure 3A), and EEG showed that the EEG activity was relatively depressed (low amplitude and lack of changes). Auditory brainstem evoked potentials suggested hearing impairment, later EEG showed epileptiform EEG changes, and head MRI showed brain atrophy (Figure 3B). Case 2 died due to uncontrolled status epilepticus and ineffective treatment at the age of 3 years and 3 months (2022.8.10), and was diagnosed as type II GD.

#### 2.1.6. Ophthalmic involvement (ophthalmology)

There are relatively few studies on ophthalmic involvement in GD. Some patients may have symptoms such as retinopathy, corneal opacity, uveitis, pinguecula and conjunctival thickening, abnormal eye movements, and strabismus (18, 19). No ophthalmic involvement was found in both children in this study by Slit-lamp microscopy, fundus photography, and other ophthalmic examinations.

#### 2.1.7. Involvement of the cardiovascular system (cardiology)

Cardiac involvement in GD is extremely rare, and only case reports have been reported. It mainly occurs in subtype c of type III GD, which occours in the homozygous D409H genotype (20). Cardiomyopathy and valvular disease are the manifestations of involvement, and there are also structural and functional abnormalities of the right heart secondary to pulmonary hypertension, which can be clinically manifested as cardiac failure, arrhythmia, and increased incidence of thromboembolic events with atrial enlargement (21). In this study, both children were evaluated by ECG, cardiac ultrasound, and myocardial enzyme spectrum, and no cardiac involvement was observed.

#### 2.1.8. Involvement of the respiratory system (respiratory medicine and PICU)

Gaucher cells may directly infiltrate lung parenchyma and pulmonary vessels to cause pulmonary hypertension or interstitial lung disease. Pulmonary function examination mainly shows the decline of diffusion function, accompanied by varying degrees of restrictive ventilatory dysfunction. Imaging may show signs of pulmonary interlobular septal thickening, ground glass-like changes, reticular nodule infiltration, air retention and bronchiectasis (22, 23). Due to the obvious symptoms related to the involvement of extrapulmonary organs, respiratory symptoms are easily overlooked, which should be paid attention to by clinicians. GD patients with p.L483P gene mutation are prone to respiratory system involvement (24). In case 1, there were no abnormalities in the monitoring of oxygen saturation, determination of serum markers of pulmonary fibrosis, pulmonary function and pulmonary CT examination from initial diagnosis to follow-up. In case 2, pulmonary CT showed a small amount of inflammation in the upper lobes of both lungs at the first visit, pulmonary function test indicated mild mal-ventilation, and serum markers of pulmonary fibrosis showed elevated collagen IV. Pulmonary inflammation was aggravated several times during treatment and follow-up, which was improved by anti-infection and supportive treatment. It may suggest that ERT does not improve the occurred lung damage.

#### 2.1.9. Renal involvement (nephrology)

Different from other storage diseases, renal involvement in GD is rare, and clinical manifestations include proteinuria, microscopic hematuria, renal tubular dysfunction, and renal insufficiency or even failure (3). Both children in this study were evaluated by routine urinalysis and renal function tests, and no renal involvement was observed.

#### 2.1.10. General surgery

GD patients with ischemic splenic infarction due to enlarged spleen may develop acute abdominal pain, fever, and inflammation around the spleen (3). For those with giant spleen or hypersplenism, splenectomy can significantly improve clinical symptoms, reduce anemia and bleeding tendency, and improve developmental status (25). There was no obvious anemia and bleeding tendency caused by hypersplenism in both children in this study, and no surgical intervention was performed.

### 2.2. Therapeutic intervention

Specific treatments for GD mainly include ERT, hematopoietic stem cell transplantation (HSCT), substrate reduction therapy (SRT), molecular chaperone therapy and gene therapy. Among them, ERT can specifically supplement the enzyme lacking in the patient's body and reduce the storage of glucocerebroside in the body, which is the standard treatment for GD (26–28). The mechanism of action of SRT is to inhibit substrate formation and directly reduce substrate accumulation in cells. Currently, SRT is only applicable to adults, not children (29). Gene therapy, which is still in the clinical research phase and needs to be evaluated.

As the first-line treatment of GD, ERT can shrink the volume of the liver and spleen, improve anemia, thrombocytopenia, relieve bone pain, maintain normal growth and development, and improve quality of life (26–29). However, ERT drugs are macromolecules that cannot penetrate the blood-brain barrier and cannot improve neurological symptoms (15).

In this study, case 1 was diagnosed as type I GD at the beginning of the disease after multidisciplinary discussions. Due to high risk factors such as severe growth retardation and skeletal imaging changes (30), cerezyme was given intravenously at 60 U/kg, once every 2 weeks. The volume of the liver and spleen was significantly reduced during the 2.3-year treatment and follow-up. Anemia and thrombocytopenia gradually recovered within 6 months of treatment, and three types of hemocytes remained within the normal range. The indexes of physical development gradually improved from severe growth retardation to mild level, and the monitoring indicators of bone metabolism and glucose metabolism had always been within the normal range. No progressive damages to bones and no fractures occurred. The nervous system, cardiovascular system, respiratory system, renal system and eyes were not damaged. The quality of life improved significantly.

Case 2 could not be classified because of mild neurological involvement in the early stage. After multidisciplinary discussion, considering the existence of high-risk factors such as growth retardation, skeletal imaging changes, and neurological symptoms, cerezyme was given intravenously at 60 U/ kg, once every 2 weeks, and nutritional support was given at the same time. The volume of the liver and spleen was significantly reduced during the treatment and follow-up. Anemia and thrombocytopenia recovered gradually within 6 months of treatment. However, the indexes of physical development did not improved, and the growth retardation gradually progressed from moderate to severe level. There were also progressive damages to the bones, but no fractures occurred. The damages to the central nervous system progressed rapidly with frequent convulsions and brain atrophy. The patient died at the age of 3 years and 3 months after poor anti-epileptic treatment. There was mild mal-ventilation in the lungs at the beginning of the disease, and pulmonary inflammation was aggravated several times during the treatment and follow-up, and anti-infective treatment was given. The cardiovascular system, renal system and eyes were not damaged. According to the early involvement, rapid progression of the nervous system, and multiple organ damages, the patient was diagnosed as type II GD.

## 3. Conclusion

GD is a disease involving multiple systems, requiring multidisciplinary cooperation in the diagnosis and treatment process. In addition to the above clinical departments, more departments such as clinical laboratory, imaging department, genetic testing, pathology, pharmacy and nutritional department are needed to accurately diagnose and treat, improve the prognosis and quality of life, and reduce the risk of skeletal and other systemic disabilities (31). In addition, there is currently no effective treatment for children with type II GD.

## Data Availability

The original contributions presented in the study are included in the article/Supplementary Material, further inquiries can be directed to the corresponding author/s.
